# Reliability Analysis of Reinforced Concrete Structure with Shock Absorber Damper under Pseudo-Dynamic Loads

**DOI:** 10.3390/ma15072688

**Published:** 2022-04-06

**Authors:** Chun-Chieh Yip, Jing-Ying Wong, Mugahed Amran, Roman Fediuk, Nikolai Ivanovich Vatin

**Affiliations:** 1 Department of Civil Engineering, Universiti Tunku Abdul Rahman, Cheras, Kajang 43000, Selangor, Malaysia; 2 Department of Civil Engineering, University of Nottingham Malaysia, Semenyih 43500, Selangor, Malaysia; jingying.wong@nottingham.edu.my; 3 Department of Civil Engineering, College of Engineering, Prince Sattam Bin Abdulaziz University, Alkharj 16273, Saudi Arabia; 4 Department of Civil Engineering, Faculty of Engineering and IT, Amran University, Amran 9677, Yemen; 5 Polytechnic Institute, Far Eastern Federal University, 690922 Vladivostok, Russia; fedyuk.rs@dvfu.ru; 6 Peter the Great St. Petersburg Polytechnic University, 195251 St. Petersburg, Russia; vatin@mail.ru

**Keywords:** scaled model, damper, dynamic response, seismic, reliability analysis

## Abstract

Past historical earthquake events from neighbouring countries have been proven to be disastrous. Building in the aftermath of an earthquake may reduce structural reliability, posing risk upon re-occupation of the building. Shock absorber viscous dampers were installed on a specific structure storey that could reduce the spectral acceleration and storey-drift caused by an earthquake. The research object is a low-rise, three-storey, reinforced concrete (RC) structure. This study aims to identify the dynamic response of the scaled RC structure with and without attached dampers and performs structural reliability of the tested model under the excitation of Peak Ground Acceleration (PGA) of 0.1 g to 1.0 g with a unidirectional shaking table. APIDO viscous dampers were installed parallel to the movement direction of the dynamic load test. The findings show the scaled model with attached viscous dampers reduces spectral acceleration and storey drift by 9.66% and 4.85%, respectively. Findings also show the change of the structural behaviour from single curvature to double curvature due to the increase in seismic structural resistance by viscous dampers. The breakthrough of this research shows that structural reliability analysis performed by the Weibull distribution function has a base shear capacity increment of 1.29% and 6.90% in seismic performance level Life Safety (LS) and Collapse Prevention (CP), respectively. The novelty of this case study building with dampers managed to increase the building’s base shear and roof shear capacity by 6.90% and 16% compared to the building without dampers under dynamic load excitation.

## 1. Introduction

Earthquakes are one of the most feared natural disasters on Earth. The peak ground acceleration (PGA) represents an object’s maximum recorded lateral acceleration during an earthquake. The unit for PGA is denoted as “g” in “m/s^2^”. Researchers widely use PGA to investigate the underground movement. The earth is generally made up of four layers: The outer crust, followed by the mantle, outer core, and inner core. The structural mantle layer is the thickest layer, with an approximate thickness of 3000 km [1]. The layer itself is semi-solid, leading geologists to believe this weak mantle part is where the Earth’s tectonic plates glide over. Stresses accumulate between the tectonic plates until they exceed their bearing stress capacity, and a shockwave is released due to the movement of tectonic plates [2]. This movement is what people usually refer to as an earthquake. When earthquakes occur underneath the ocean, a series of large waves, known as a tsunami, is created. Both earthquakes and tsunamis can cause devastating damage to cities [3].

**Figure 1:**
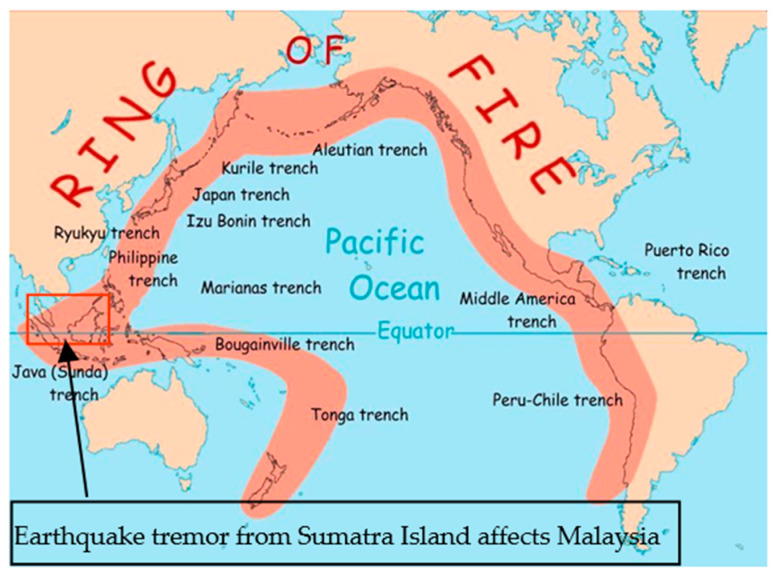
Ring of fire, covering Malaysia.

Earthquakes usually do not strike at random places but rather mostly occur in a specific region, popularly known as the Pacific Ring of Fire [4]. It is a region that stretches the length of the Earth’s tectonic plates at the Pacific Ocean, termed and described by its active volcano and earthquake activities. Almost 90% of the entire history of earthquake events occurred within this region [5]. However, it is still possible for countries outside this region to experience far-field earthquakes from neighbouring countries. Malaysia is located at the edge of the Ring of Fire, implying the country itself is not necessarily free from a seismic event. Between 1990 and 2016, 182 earthquakes with magnitudes ranging from 2.9 to 6.0 were recorded in Malaysia (see Figure 1) [6]. One of the major earthquake events that occurred on land was the Sumatra-Andaman Earthquake with a magnitude of 9.1 [7]. The event caused 68 deaths across Kedah, Penang, Perak, and Selangor. Since that event, Malaysians have started to grasp how destructive an earthquake could be without proper structural seismic design. Thus, necessary engineering standards and structural designs are required to provide the safety of structures under seismic action [8].

It is necessary to implement a versatile composite element that combines two materials that act together in resisting forces. Plain or unreinforced concrete is weak against tensile and shear stresses [9]. To overcome this issue, reinforcing steels are usually embedded in concrete during the concrete-making process, or fibre-reinforced concrete can also be added to resist seismic energy [10,11]. By allowing the concrete and reinforcement to set, a strong bond is formed between the two materials through the crystalline structure within the fibre-reinforced concrete materials. As a result, it significantly increases the concrete’s overall strength towards applied forces [12]. RC is widely used in building construction due to its cheap cost and acceptable bearing resistance towards external forces. However, its seismic resistance is still limited due to its brittle behaviour, causing the building to collapse when subjected to earthquake loading [13]. Buckling is one of the most common types of failure in reinforced concrete (RC) structures due to earthquakes and the mechanical solutions for RC failure such as deflection, for different RC elements were reported by many researchers [13,14,15,16,17,18,19,20]. Buckling occurs due to a lack of reinforcing steel within the RC, causing the RC structural element to bend. The failure mechanism of buckling can cause a soft and weak storey of the structure, where drifting of the structure occurs due to seismically vulnerable supports such as non-load-bearing wall panels that are adopted at the base of the structure [17]. A bigger section with more reinforcing steel can be used to prevent such failures to prevent RC structures from collapsing easily [21]. 

In addition to that, seismic resistance designs such as bracing and dampers can also be adopted. Bracing is one of the most common types of construction used to provide structural stability and increase a structure’s resistance against lateral seismic loading [21,22]. Bracings are usually arranged in a diagonal manner and are nominally pinned, connected between the beams and columns of the structure [13,22]. There are two types of bracing: Eccentric bracing and concentric bracing. A framed structure is referred to as eccentrically braced when one or both ends of the diagonal brace members of the frame structure do not meet and join at the endpoints of another framing member [18,23,24]. It is stated that when the ends of the diagonal brace members of the frame structure do meet and join at the endpoints of another framing member, the frame is termed concentrically braced. Both types of bracings have been proven to reduce the sideways storey drift of a structure, and 87% and 48% of the maximum lateral storey drift was reduced at the topmost floor of a tested structure with concentric and eccentric bracing, respectively [25]. However, these types of seismic resistance designs are not very efficient for a severe earthquake event as the frames are prone to large lateral displacements. Thus, they are usually used along with another type of seismic resistance system known as dampers. As with bracings, dampers are used to control the structural damages through the dissipation of seismic energy [26]. Examples of dampers include friction dampers and viscous dampers. Friction dampers consist of a few specially treated and designed steel plates that slide across each other to develop friction when a seismic load is applied. Through the principle of friction braking, the friction damper converts the seismic kinetic energy impacted upon the structure into heat energy via the rough motion between plates [27]. A similar mechanism can also be seen in the viscous damper, where fluid flows back and forth through the different sizes of the chamber, causing the seismic energy to dissipate due to head loss [28]. By adopting these dampers, structural damping ranging from 20% to 50% can be achieved [29]. Through the combination of both dampers and bracing, a higher reduction in seismic damage can be achieved. 

Dynamic tests such as the shaking table test are used to evaluate the effectiveness of such resistance design. The shaking table test is one of the pseudo-dynamic approaches used in assessing the structural behaviour under seismic loads. It considers the dynamic effect of the seismic event in terms of seismic loading, shear loading, and primary waves during the analysis, thus reflecting the real earthquake effect. The results obtained from this test are more promising than the conventional pseudo-static test [30]. The degree of freedom (DOF) of the shaking table also affects the accuracy of the result; a higher DOF yields a more accurate result. According to research conducted by [31], a six-DOF shaking table can control and minimise the off-axis input and, thus, cause less error while experimenting. However, six-DOF testing requires an accurate description of the time series and locations of accelerometers to calculate the rotational inputs to the shaking table. With this, a higher performance design with lower conservatism and risk can be facilitated by the six-DOF model compared to the one-DOF shaking table [32].

**Figure 2:**
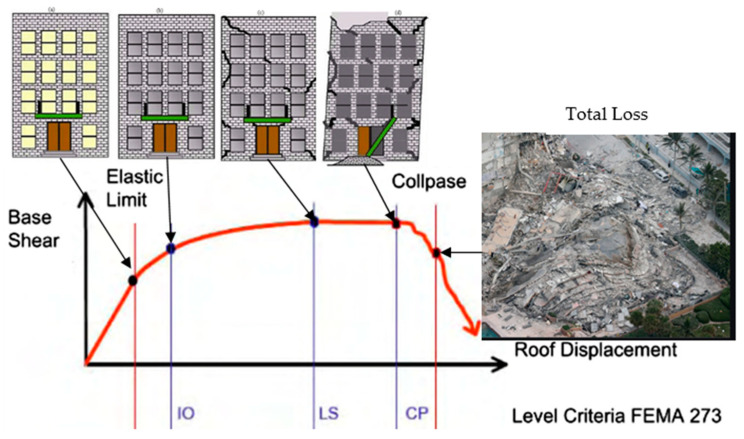
Graph of building response to member response.

There are generally four types of seismic performance assessment models to evaluate the seismic performance level of a structure: Linear Static Procedure (LSP), Nonlinear Static Procedure (NSP), Linear Dynamic Procedure (LDP), and Nonlinear Dynamic Procedure (NDP). These procedure methods require different parameters and working conditions. Hence, prior to conducting the assessment, information such as the location and environment of the site, the size and layout of the structure, the strength and stiffness of the structure, the number of occupancies, as well as the location, properties, and types of finishes, and the non-structural system must be known, so that the most suitable model can be adopted to acquire accurate results [33]. Through evaluation, the performance level of a building shown in Figure 2 can be categorised quantitatively and qualitatively into one of the four discrete standard levels, namely:

▪ Operational (O).
▪ Immediate Occupancy (IO).
▪ Life Safety (LS).
▪ Collapse Prevention (CP).

General qualitative and quantitative descriptions of each seismic performance level are shown in Table 1 [33] to facilitate this research in identifying the building performances at each level [34]. The current research gap in accessing the reliability of a seismic performance structure with and without viscous dampers equipped is still absent. This paper intended to close the research gap through a reliability analysis of the seismic performance of a building with and without the viscous dampers equipped. Besides, the seismic performance of the mechanical shock absorber is very limited as well. Therefore, the following seismic performance level published by the Federal Emergency Management Agency (FEMA) from the United States will be adopted to assess the structural seismic performance. This could lead to the discovery of the effectiveness of using shock absorber dampers as structural dampers rather than the friction or viscous dampers introduced earlier by other researchers. The novelty of this research is to unveil the reliability of mechanical shock absorbers in dissipating the seismic energy for buildings as another alternative solution to ensure the safety of the building.

As mentioned, LSP, also known as the static lateral force procedure, is an analytical method in which the seismic responses are determined through the application of a series of static forces in each principal horizontal axes, namely x- and y-axes, of a structure with linear elastic behaviours in terms of the stiffness and equivalent viscous damping value. The magnitude of the lateral design forces is chosen with the intention that, when exerted upon the linearly elastic structure, a displacement design that has a value nearing the maximum displacement expected during the analysis subjected to seismic loading is produced. For LSP, the displacement response shows the damage suffered in the nonlinear range of the building’s response to force; in the nonlinear range, even a small change in the force will result in large structure displacement. Thus, if the structure responds inelastically to the seismic design load, the internal forces developed in the structure will be less than those calculated based on the lateral design forces [35]. 

LDP is similar to LSP, but instead of evaluating the seismic performance of the structure using a static seismic loading, LDP takes the dynamic behaviour of the seismic loads into design consideration where multidirectional seismic responses are accounted for in the concrete behaviour after an earthquake. This study shows the crack propagation speed, the first crack initiation stress, the coalescence stress, the compressive strength, and the ultimate strain increase related to the loading rate. The dynamic loading rates influence the position of the crack and propagation direction with the cracking length [36]. Thus, the results predicted by LDP can be said to be more accurate than LSP as the earthquake’s motion can be properly represented during the design analysis. In addition to that, LDP is better suited for predicting the distribution of structural demands with irregular mass distribution, stiffness distribution, and geometries. The results yielded will be more promising than those produced by LSP. There are generally two types of LDP: The time history method and the response spectrum method. Both methods are only feasible in structures with linearly elastic behaviours, and both working principles revolve around Newton’s Second Law of Motion equation [37]. The time history method assesses and calculates the structural dynamic response of the structure under earthquake excitations described by ground acceleration movement at regular time intervals [38]. The response spectrum method is an elastic dynamic analysis used to assess the dynamic responses of all modes of structure, which eventually contributes to the overall structural response [39].

NSP, more commonly known as pushover analysis, is used to evaluate a structure’s seismic performance based on parameters such as global drifting, inter-storey drifting, and inelastic elements deformation. It is an assessment model that is further derived from the conventional LSP, and thus, only the horizontal seismic responses are resolved during the analysis [40]. NSP estimates the seismic load and structural deformation, which accounts, in an approximate fashion, for the internal forces redistribution occurring within the structural system when it is subjected to a monotonically increasing horizontal load, which represents the inertia forces during a seismic event. However, NSP cannot accurately account for the changes in the dynamic behaviour of a structure in the analysis process due to its degradation of the structure in terms of stiffness. Hence, LDP is sometimes used alongside NSP to determine the adequacy of design; this not only provides verification of the analysis results, but also improves the knowledge obtained by performing both procedure methods [41].

NDP is very similar to NSP in terms of the basis, modelling steps, and acceptance criteria. The main difference that distinguishes them is that in NDP, a nonlinear time history analysis is used to calculate the seismic responses; the design displacements for NDP are identified through the dynamic analysis, adopting past historic ground motions instead of using the target displacement as in NSP. It evaluates the seismic responses of a structure described in underground motion records. The dynamic seismic actions gradually affect the building with time intervals, Δ*_t_*, and the constraining motion equations are solved using a direct integration procedure. The seismic responses are evaluated for a span of time with a series of incrementally short times. The general equation of motion is as shown as Equation (1). The vibration of the base movement of the structure yields a complete seismic response, such as displacements and stress resultants, thus leading to tremendous amounts of data obtained through NDP [42].

MU (t) + CU (t)+ KU (t) = −MU_g_ (t) (1)

where M is the mass matrix, C is the damping matrix, K is the stiffness matrix, U is the displacement vector, U is the velocity vector, U is the acceleration vector, and U_g_ is the ground acceleration vector

After conducting a seismic performance assessment, the output from the assessment model must be carefully selected to interpret the results accurately. The outputs from the procedure model are divided into two types: Actions and deformations. Action outputs can be represented both locally and globally. Generally, local actions include the output of stress and strain of the discretised system. The normal stress, shear stress, and the combinations of both stresses, known as the equivalent stress, can be identified based on the geometry of the applied load types and the discretisation adopted by the tested structure. On the contrary, global actions are generally correlated with internal actions such as axial forces, shear forces, bending moments, and torque. Deformation parameters generally provide clearer insight into the damage inflicted upon the structure subjected to seismic load than the action parameters and can be represented both locally and globally. Local deformation concerns the structural element’s normal strain and shear strain, which can be obtained through detailed geometric discretisation of the building. These values are used to estimate the chances of local buckling of the structural cross-section and its reinforcing bars; normal strain is determined to identify the likelihood of shear yielding and buckling of the structural elements, while shear strain is determined to monitor the curvatures, steel yielding, and concrete crushing in the structural elements of the building [43,44,45]. On the contrary, global deformations parameters such as inter-storey displacement can be used to identify the occurrence of structural damages [46].

Another type of unique output from the assessment model is the mode shape. It generally shows the shape of deformation that the structure would experience when subjected to vibration at its natural frequency. Mode shapes have no scientific units associated with them, hence making them not viable for use as a quantitative assessment. Rather, it is more suitable to use them in qualitative evaluation when the structure is subjected to dynamic loads. Mode shape has three fundamental modes of oscillation, namely:

- Simple translation at the x-axis.
- Simple translation at the y-axis.
- Rotation about the z-axis.

The sum of every mode response shows the overall response of the building. Mode shape is an independent variable that is not affected by the force applied to the structure. However, mode shape varies when civil construction materials properties such as weight, height, stiffness, and damping value of the structure change [47]. 

Once the outputs are properly identified, the structural reliability can be analysed. Structural reliability analysis approximates the probabilities of the structural limit state under adverse loading such as seismic loading for an expected time span of use. It is frequently correlated with the term ‘safety’ and can offer a probabilistic expression. However, due to uncertainties present in an earthquake scenario, structural reliability analysis becomes more complex. The exact figure of the probability of structural failure is unachievable for unpredictable events. On a more positive note, when uncertainties are taken into analysis consideration, the analysis process will yield a more accurate result. Though extra parameters may be introduced, all of these can eventually be assessed through numerical analysis [48]. One of the most widely adopted models to evaluate reliability is the Weibull Distribution Model. In reliability engineering, the Weibull distribution attempts to make an approximation of the lifespan of a certain object or product by manipulating the statistical distribution of life results from a set of data acquired from the population. In layman’s terms, the Weibull distribution is used to foresee the characteristics of the critical life cycles at a specific time. The practicality of the Weibull distribution is valued in this research because of its high versatility that accounts for the characteristics of other types of distribution through the application of parameters in the model function. Generally, there are two Weibull Probability Density Function (PDF) types: Two-parameter Weibull and three-parameter Weibull. The general equation of the reliability of two-parameter and three-parameter Weibull distributions, *R*(*t*), is shown as Equations (2) and (3) [49].

(2) $$R\left(t\right)=e^{-{\left(-\frac{t}{\alpha }\right)}^{\beta }}\;$$

(3) $$R\left(t\right)=e^{-{\left(-\frac{t-\gamma }{\alpha }\right)}^{\beta }}.\;$$

where *α* is the scale parameter, *β* is the shape parameter, and *γ* is the location parameter.

**Figure 3:**
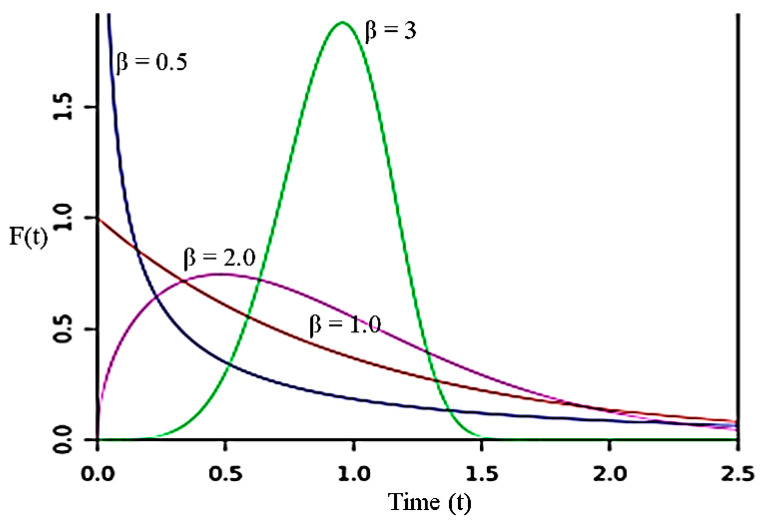
Weibull PDF curve with varying *β* values.

In the Weibull distribution, *β* determines the shape of the distribution curve to identify the lifetime behaviour of the tested specimen. Thus, this is why *β* is also known as the Weibull slope. Generally, *β* is divided into three ranges, and each range has a different effect on the Weibull PDF. If the value of *β* is less than 1, then the failure rate decreases over time [49]. This could potentially indicate early-life failure. It is found that if the value of *β* is larger than 1, the rate of failure increases over time. This could potentially indicate the issue of premature wear. Lastly, if the value of *β* is equal to 1, it shows that the rate of failure is constant over time, which can be represented with the exponential distribution [50] as shown in Figure 3.

There are several methods used to estimate the parameters of a Weibull distribution: The Maximum Likelihood Estimator (MLE), Least-Squares Method (LSM), Method of Moment (MOM), etc. These methods are generally considered better in terms of parameter approximation than the conventional linear regression method, as numerical analyses are used to converge and minimise the error in analysis. However, MLE, LSM, and MOM have been proven to be more time consuming than estimating the parameters through regression and the intercept of the reliability curve generated by equations, as numerous iterations are required to ensure an accurate result. Even so, the simple linear regression method may also be used as it is appropriate in the approximation of Weibull parameters. Though the accuracy may be inferior to the estimated parameters computed by MLE, LSM, or MOM, it is still feasible [51]. 

## 2. Design and Methods

A one-bay, 3-storey, low-rise RC structure was built to evaluate the seismic performance of an RC structure with seismic resistance. This project started with the modelling of a scaled structure followed by the shaking table test in the laboratory to understand the impact of earthquake load on the structure. A properly constructed model can aid in demonstrating the dynamic behaviour and failure mode of the structure. A scaled-down structure is usually used to save costs in model construction. Nevertheless, researchers can acquire similar results as from full-scaled models using the scaled-down models. The acquired results were then further analysed for their structural reliability. A globally accepted structural reliability analysis method introduced by Dolas, Jaybhaye, and Deshmukh (2014) [49] was adopted in this research to assess the performance levels of a structure after an earthquake ensues.

### 2.1. Materials Preparation

Generally, concrete is a mixture of cement, water, fine aggregates, and coarse aggregates. To produce a good reinforced concrete structure, crucial work such as good compaction during the placement of the fresh concrete and fine workmanship in screeding and curing after the placement is important. In this research, the type of cement used was Ordinary Portland Cement (OPC), also known as Type I cement (Hume Portland Cement, Selangor, Malaysia). The physics parameters and chemical composition of the OPC used in this research are shown in Table 2 [52]. The physical properties of OPC contain base elements such as lime, silica, alumina, iron oxide, sulphur trioxide, etc., that can react with H_2_O to bind the coarse and fine aggregates to form a crystalline structure. OPC is commonly used for general construction when there is no exposure of sulphates within the environment, especially in the soil or groundwater. Sulphate-Resisting Portland Cement should be used if sulphates are found in the soil. Since the research focuses on the seismic resistance of the structure, external factors such as soil properties should be omitted. This research used materials such as coarse aggregates (Kajang Granite Quarry Sdn Bhd, Selangor, Malaysia) and fine aggregates (Kajang Granite Quarry Sdn Bhd, Selangor, Malaysia), with sizes of 5 mm and 600 μm. Crushed 5 mm granite coarse aggregates (UTAR Sg. Long, Selangor, Malaysia) with a compressive strength of 21 N/mm^2^ were used in this model construction, purchased from the local quarry from Kajang Granite Quarry Sdn Bhd. During the preparation of both coarse and fine aggregates, sieves with a standard hole size (UTAR Sg. Long, Selangor, Malaysia) were used to filter out unwanted debris such as organic matter that may influence the material strength. Tape water (UTAR Sg. Long, Selangor, Malaysia) acts as a binding agent, which creates the chemical reaction from the cement powder to join the aggregates together to form concrete. In addition to that, tap water is free from impurities such as suspended solids, organic matter, dissolved salts, etc. The mixing procedures were carried out in accordance with BS 3148.

In addition to those common materials, Masterglenium Sky 8808 plasticizer (UTAR Sg. Long, Selangor, Malaysia) was also used to reduce the water-to-cement (w/c) ratio to produce high strength concrete. Once materials are prepared, the next step is to design a suitable cement mixture to form Grade 35 concrete. The trial-and-error method was used to identify the optimal ratio of raw materials to produce 9 cylindrical concrete samples with satisfactory performance in terms of compressive strength. After all rounds of the trial, the optimal ratio of raw materials was obtained and is shown in Table 3. In addition, Table 4 tabulates the compressive strength test of the 7-day-cured cylindrical sample in accordance with the ASTM C109 standard. Note the cylindrical concrete samples were cast using the optimal design mix proportions. 

### 2.2. Model Construction

**Figure 4:**
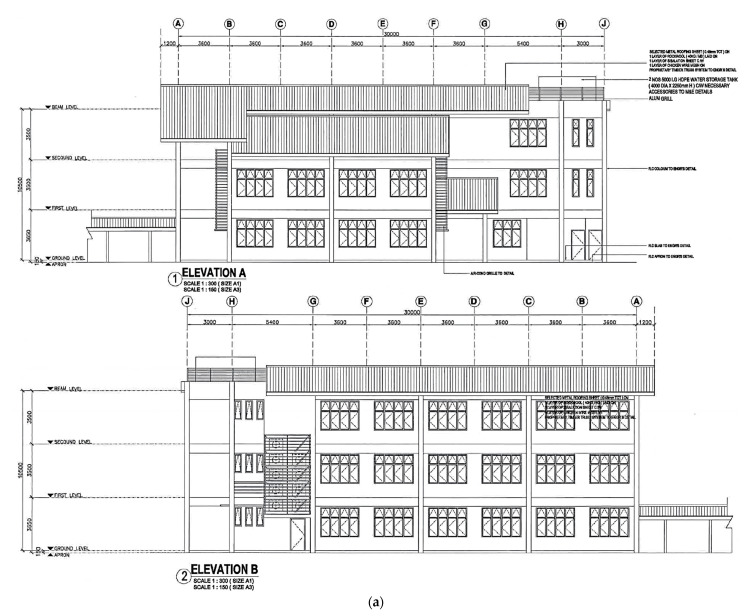

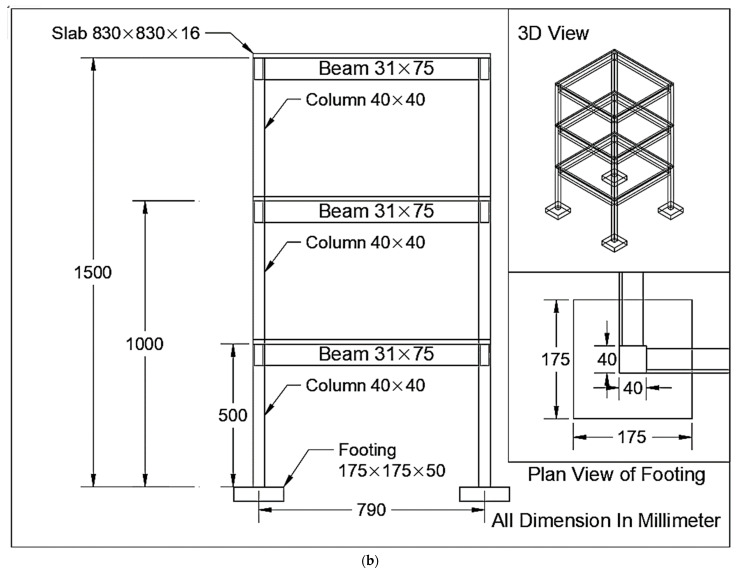
(**a**) Elevation view of full-scale building; (**b**) general layout of scaled RC structure.

The technical documents, such as full-scale building Elevation A and Elevation B, are shown in Figure 4a. This is a school building where the technical drawings and designs documented by (Public Work Department) local authorities are owned by Wonderful Engineering & Construction Sdn. Bhd. Due to the frequent tremors from neighbouring countries, the building owner worried it might pose certain risks or building damage due to the tremors, which led to this research study. The building owner proposed the study of the parameter frame of the building highlighted in the red box, 7.2 m in span and 3.5 m in height. The first limitation occurs where the structure has a beam size of 31 mm in width by 75 mm in depth and a column size of 40 mm in width by 40 mm in breadth with a centre-to-centre beam span of 790 mm, utilising a scale factor of 1:9. Moreover, 4 pad footings were constructed at each of the columns with dimensions of 175 mm in width by 175 mm in breadth by 40 mm in height for fastening the shaking table platform. The slabs covering the first, second, and topmost floors of the structure have dimensions of 830 mm by 830 mm and is 16 mm thick. The second limitation concerns the alternate scale factor of 1:7, which generated a total storey height of 1500 mm with a 500 mm inter-storey height. The different scale factors accommodate the available space of the shaking table machine and the availability of materials in the hardware store to fabricate the scaled buildings. The utilisation of scaled factors has common agreement among researchers [53,54,55], showing promising accuracy of the results.

Figure 4b illustrates the general layout of the 1-bay, 3-storey RC structure. The construction process of the structure started with the formwork, followed by the fabrication of reinforcing steel at the ground level. As pad footing acts as the base support for the structure, it is crucial to start the construction there. The diameter of the steel reinforcement used for pad footings was 3 mm. The fresh concrete paste was poured into the formwork after the rebar was bent and placed as designed. Prior to that, a layer of plastic wrap was placed inside the formwork to prevent adhesion between the hardened concrete and formwork during the demoulding process. After 1 day, the formwork was removed and was left to cure for 7 days. Once the pad footing was cured, formwork construction of the columns on the ground floor was performed on top of the pad footings. As with the pad footing, the steel bar diameter was 3 mm. As column structures are slender, shear links were required to ‘tie’ the vertically placed steel bar together. For the ground level columns, the shear link was placed every 40 mm beginning from the top of the column. With everything ready, the fresh concrete mix was poured into the wrapped formwork with embedded steel bars and left for 1 day before performing the demoulding process. Then, it was left to cure for 7 days before constructing the beam structure. In the meantime, the formwork for the beam structure on the first floor was completed. The diameter of the steel reinforcement was also 3 mm. Regarding the shear link placement, the link was placed at every 32 mm. Once the steel was placed into the formwork, the fresh concrete mix was then poured to cast the beam structure. Again, the structure was left for 1 day for hardening and 7 days for curing. Lastly, the casting of the slab on the first floor was performed. As with other RC elements, the formwork must be constructed. Steel reinforcement of the slab 3 mm in diameter was fabricated and shaped. After everything was ready, the slab was cast and left for 1 day for hardening and 7 days for curing. Once the slab structure was cast, the casting of the structure at the second storey was performed similarly to the aforementioned method and procedure. After constructing the second storey, the third storey of the structure was then cast. Note that the steel type and steel arrangement, as well as the dimensions of the RC structures, were all the same. Hence, the process of constructing the formwork, steel fabrication, and, lastly, the concrete casting of the structural elements was repeated until the topmost floor was completed.

### 2.3. Experimental Setup

After the RC structure was built and cured, the whole structure was then carefully lifted and placed on top of a shaking table (Kobe University, Kobe, Japan) with the aid of a pallet stacker truck. The shaking table used for this project was a 2 m by 2 m shaking table operated by a levelled platform (UTAR Sg. Long, Selangor, Malaysia) and an actuator (Kobe University, Kobe, Japan). The motor-driven actuator was installed along with the levelled platform, and the shaking was performed via the rotation of the motor. Other components such as the signal controllers, device-controlling software, and motor-controlling software were also installed to achieve satisfactory operation (Kobe University, Kobe, Japan). To drive the motor, 420 V of alternating current must be transformed to 200 V, which is then used to supply power to the motor; it is operated by voltage supply and signal transmission. Alternatively, the drive unit is also capable of providing the horizontal shaking motion by simply receiving the signal from an end-user; the digital signal of the displacement and the frequency of the shaking motion input by the end-user into the motor-controlling software (MotCtlProg, Version 1.1, Kobe, Japan) is converted into an analogue signal by a digital-to-analogue converter. Ultimately, the signal will be sent to the motor and is now ready for shaking. The shaking movement of the shaking table is directly associated with the combined effect of the input displacement and frequency by the end-user; with a high shaking frequency, a lower magnitude of displacement must be applied, and vice versa. The seismic load exerted upon the shaking table will also be changed by simply changing the motion setting in terms of frequency and displacement. 

**Figure 5:**
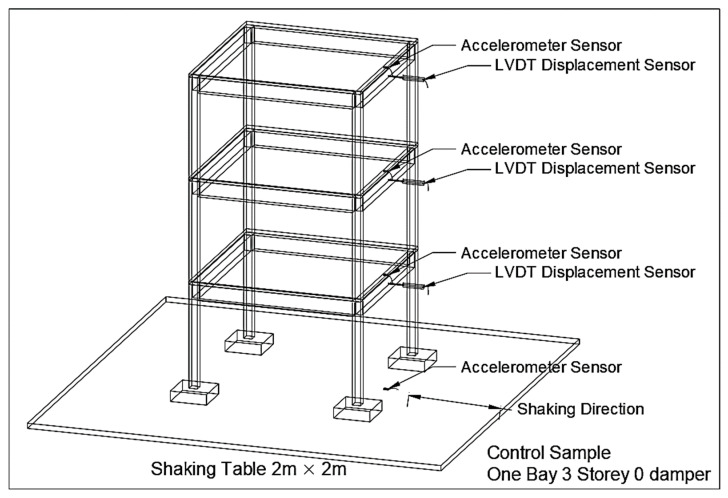
Placement of accelerometer and LVDT.

Figure 5 shows the placement of transducers for data-recording purposes; an accelerometer (Tokyo Sokki Kenkyujo, Tokyo, Japan) and a linear variable differential transformer (LVDT, Instron Malaysia Sdn. Bhd, Selangor, Malaysia) were mounted on each floor of the structure. The transducer was then connected to a data logger to record the results. The type of accelerometer and LVDT used was manufactured by Kyowa Electronic Instruments and Tokyo Sokki Kenkyuj from Tokyo, Japan. 

The accelerometer is used to quantify the acceleration and seismic vibration of the structure when prone to dynamic loading. In addition, LVDT converts the mechanical elongation or displacement into the corresponding difference in electrical resistance, inductance, or capacitance. The changes in such electrical parameters are proportional to the strain. The data logger used for this shaking table was the TML Data Logger DRA-30A (Version 30A, Tokyo Measuring Instruments Laboratory Co., Tokyo, Japan). It can convert analogue signals, including acceleration and displacement magnitude, into digital values at high speed and store these data in the internal memory or transfer them to a computer. The logger itself contains 30 channels; 30 transducers can be used simultaneously, and the data memory of each channel is 112,000 words. 

**Figure 6:**
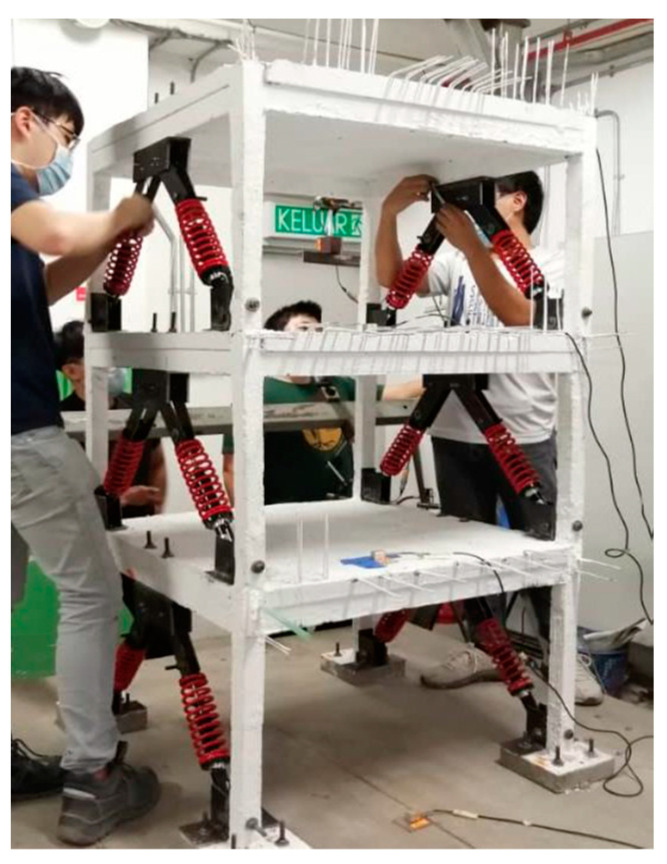
Arrangement of viscous dampers.

Once the model is in place and the sensors are mounted, the shaking begins. Prior to that, the shaking table was calibrated by placing approximately 175 kg (equivalent to the mass of the RC model built) onto the shaking table with attached accelerometers. The table was then shaken until the accelerometers showed the desired output of the PGA amount of 0.1 g to 1.0 g. The corresponding settings of the displacement and frequency of the shaking at the desired PGA were then recorded and used for the actual shaking of the RC structure. Once the input data, in terms of frequency and displacement, were acquired, the shaking of the RC structure started by simply inputting the previously acquired displacement and frequency information into the MotCtlProg software. The shaking of the structure started at 0.1 g, and the incremental intervals between each vibration were 0.1 g each. At each intensity stage, the shaking was maintained for 20 s before proceeding to the next level of vibration. The test was then ceased until the shaking table reached 1.0 g. After shaking the RC structure without the dampers attached, the dampers were installed on the same RC structure. Figure 6 illustrates the arrangement of the dampers. 

There were 12 dampers used, with 4 dampers on each storey. The dampers used were APIDO (Kee Kee Motor (KKM) Sdn Bhd, Selangor, Malaysia), which is a type of adjustable gas mechanical shock absorber that uses methyl silicone oil as the friction medium. The dampers on the side, which were parallel to the movement of the shaking table, were installed in an inverted V arrangement. Once the dampers and transducers were all in place, the shaking test started at 0.1 g and was maintained for 20 s before increasing it by 0.1 g to bring the intensity to the next level. The shaking of the structure with dampers once again ceased when it reached PGA 1.0 g. The digital values of the displacement and acceleration were then saved in the computer and used for further analysis.

### 2.4. Reliability Analysis

Structural reliability analysis of the tested RC model was conducted using the Weibull Distribution Model. The reliability of the structure was quantified based on the base shear of the tested RC structure. The base shear of the structure can be obtained by simply multiplying the acceleration spectra obtained from the accelerometer by the storey mass of the structure. After obtaining the base shears at different PGA intensities, the base shears were then arranged in ascending order and assigned a ranking number, where Rank 1 was the lowest base shear while Rank 11 was the highest. The base shear’s median rank (*MR*) was then calculated using Equation (4) based on their respective ranking. From the *MR* calculated, it was then converted to ln form using Equation (5) and Equation (6), while the base shear obtained was also converted into the ln form using Equation (7). Then, a line fit plot of *ln*(*ln* [1/(1 − *MR*)]) against ln (Shear) was plotted using the Microsoft Excel Regression Model.

(4) $$MR=\frac{\left(rank-0.3\right)}{\left(\sum^{\;}rank+0.4\right)}.\;$$

(5) $$Conversion=\frac{1}{1-MR}.\;$$

(6) $$Conversion=In(In(\frac{1}{1-MR})).\;$$

(7) Conversion=In(Shear). 

Then, the next step of the analysis was to determine the values of η and *β*. The value of *β* = 1.7 was obtained directly from the equation of the predicted line fit plot of *ln*(*ln* [1/(1 − *MR*)]) against ln (Shear). As for η, it can be calculated using Equation (8).

(8) $$\eta =EXP\left(\frac{-Intercept\;Coefficient}{\beta }\right)$$

Once the values of η and *β* were calculated, the final step was to compute the reliability of the tested RC structure using Equation (2). The intervals between base shears that were substituted into Equation (2) were self-defined. A smaller interval should be used to obtain a more flexible and accurate reliability plot. 

## 3. Results and Discussion

### 3.1. Comparison of Acceleration between the Model with and without Attached Viscous Dampers 

**Figure 7:**
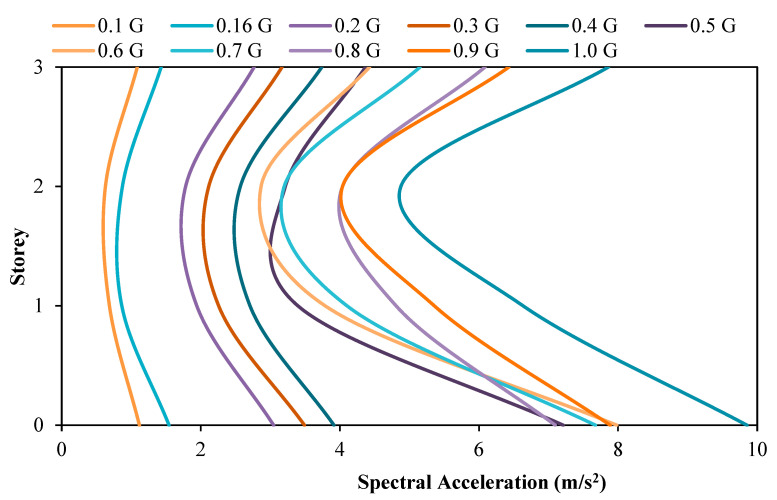
Spectral acceleration of structure without dampers.

**Figure 8:**
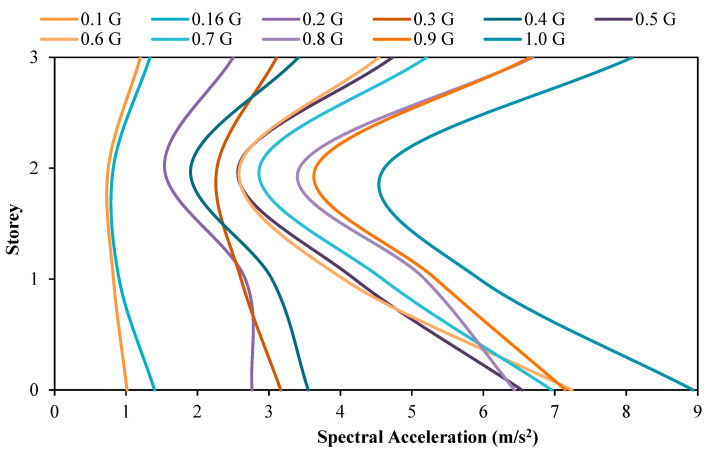
Spectral acceleration of structure with dampers.

Figure 7 and Figure 8 show the spectral acceleration response at each floor of the scaled RC structure without and with attached dampers, respectively. Generally, the relationship between the spectral acceleration experienced by the model is directly proportional to the PGA input by the shaking table. This is certainly natural, as with a higher magnitude of motion induced upon the structure, there is a higher amount of acceleration recorded, as proven by Newton’s Second Motion Law whereby the force, F, is equal to the mass, m, multiplied by the acceleration, a. Under seismic loading, F increases. The acceleration, a, of the system also increases as the relationship between these two parameters is directly proportional, as understood from Newton’s Motion’s Equation. Another trend that can be easily observed is that the spectral acceleration at the base floor is recorded to be the maximum compared to the acceleration magnitude recorded at the roof and second storey of the structure. This is also logical as the masses of the structure to return vary according to the floor of the structure, whereby a higher floor will result in a higher return of masses of the structure. Based on the principle of Newton’s Motion Equation, F = ma, the height of the structure has a direct relation to the mass, m, and is directly proportional to the lateral force, F. The experiment output shows each increment of the floor mass increases the force exerted on the base of the column as the base shear. The spectral acceleration recorded by the accelerometer at each floor, as shown in the following figures, will be used to calculate the base shear and shear force acting on the column of each respective floor.

Further observing the figures above, the roof spectral acceleration for the model with attached dampers at PGA values of 0.16 g, 0.2 g, 0.3 g, and 0.4 g is lower than the model without dampers attached. A reduction of 6.67%, 9.66%, 1.81%, and 8.67% in the roof spectral acceleration was recorded when subjected to PGA 0.16 g, 0.2 g, 0.3 g, and 0.4 g, respectively. This has successfully proven the usage of the viscous damper to reduce the amount of seismic energy induced upon the structure through the conservation of seismic energy to heat energy by moving the compressible fluid contained within the viscous dampers. In addition to that, it can also be seen that the highest reduction in the roof spectral acceleration occurred when the model was subjected to PGA 0.2 g, whereby the roof acceleration was observed to decrease from the initial value of 2.7661 m/s^2^ to a final value of 2.4990 m/s^2^ with the installation of the viscous damper. In other words, the viscous damper is deemed to be most effective when subjected to PGA 0.2 g. However, for the model subjected to PGA 0.5 g to 1.0 g, the usage of the viscous damper is ineffective as the roof spectral acceleration for the model with dampers attached is higher than the model without dampers attached. This increased amount of spectral acceleration on the rooftop may be attributed to the weight of the attached viscous damper, thus, resulting in a slightly higher roof spectral acceleration. This theory is also agreed upon by researchers [53] who conducted similar scaled-model structural testing in a shaking table test.

### 3.2. Comparison of Displacement between the Model with and without Attached Viscous Dampers

**Figure 9:**
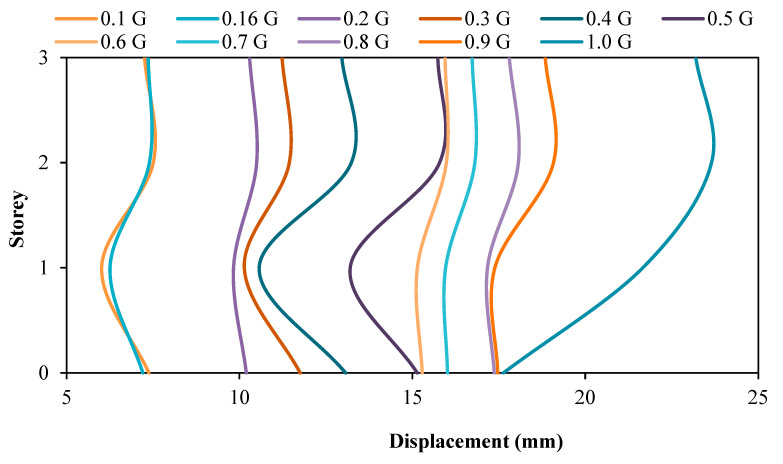
Storey drift of structure without dampers.

**Figure 10:**
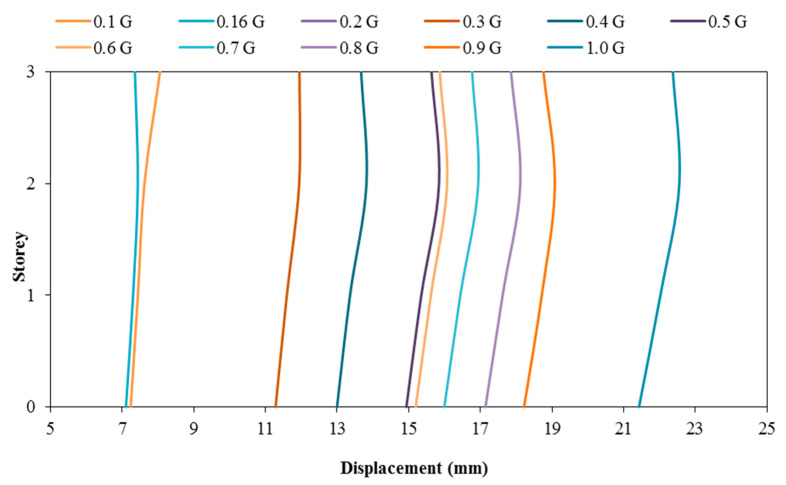
Storey drift of structure with dampers.

Figure 9 and Figure 10 show the displacement of the RC structure without and with attached dampers, respectively. Based on the displacement figures, when the PGA input of the shaking table increases, the maximum storey drift also increases. In other words, it can be said that the relationship between the storey drift of the model is directly proportional to the PGA input by the shaking table. This is true because the higher the PGA induced upon the structure, the higher the amount of seismic loading and F will be experienced by the model as written in Newton’s Motion’s Equation, F = ma. This theory is in accordance with the article written by researchers that showed when the applied force is directly proportional to the gravitational acceleration, the structural deflection will increase according to the loaded rates regardless of whether it is concerning beam or column structural elements [55]. It is found that when the seismic loading, F, increases, it is only natural that the amount of drifting experienced by the storey increases. From the same figures, it can be noted that both models with and without viscous dampers tend to experience a maximum drift at the second storey of the RC structure when subjected to PGA 1.0 g. The maximum drift at the second floor of the model without attached viscous dampers reduced from 23.650 mm to 22.555 mm, showing a 4.85% reduction in drifting when the model is equipped with viscous dampers. This has proven the usage of the viscous dampers in reducing the seismic energy transmitted throughout the structural system by converting seismic energy to heat energy with the adoption of compressible fluid within the viscous dampers. With lesser seismic energy imposed upon the structure, the drifting of the structure’s storeys will also be less. In addition to that, it can be noted that the installation of dampers in the RC model was proven to be sufficient in reducing the seismic motion upon the structure through the deflection pattern of the model; the mode shape of the model without viscous dampers attached is either Mode 2 or Mode 3, which shows double curvature when subjected to PGA 0.1 g to 0.9 g. In other words, more than one concentrated seismic load was exerted upon the structure. However, this issue does not concern the RC model with viscous dampers attached because the entire structural mode shape, when subjected to PGA 0.1 g to 1.0 g, is mode 1, which shows a single curvature that could only possibly be caused by a single concentrated seismic load. This has shown that the viscous dampers can ‘stabilise’ the drifting movement of the RC structure when subjected to low earthquake motion as well as high earthquake motion that records a PGA as high as 0.9 g, thus, reducing the deflection motion caused by an earthquake.

**Figure 11:**
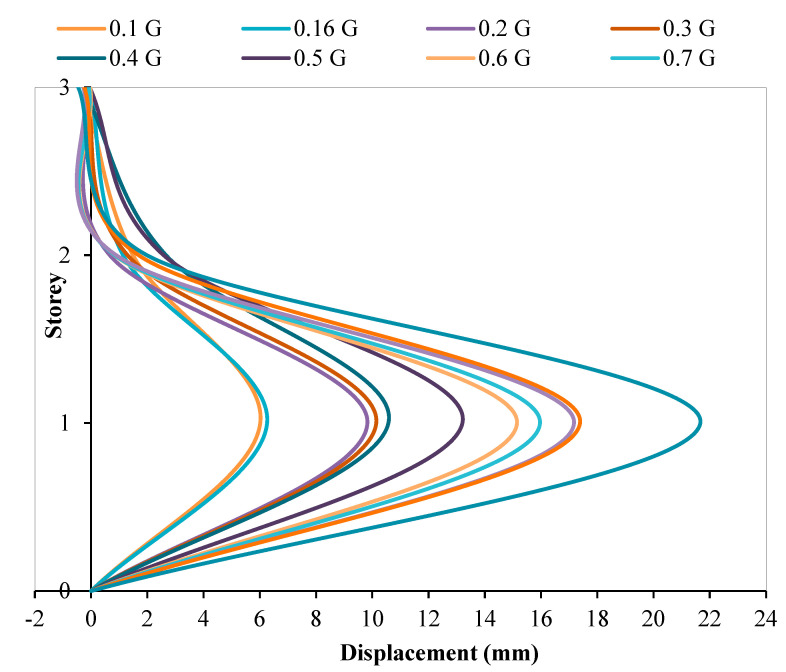
Inter-storey drift of structure without dampers.

**Figure 12:**
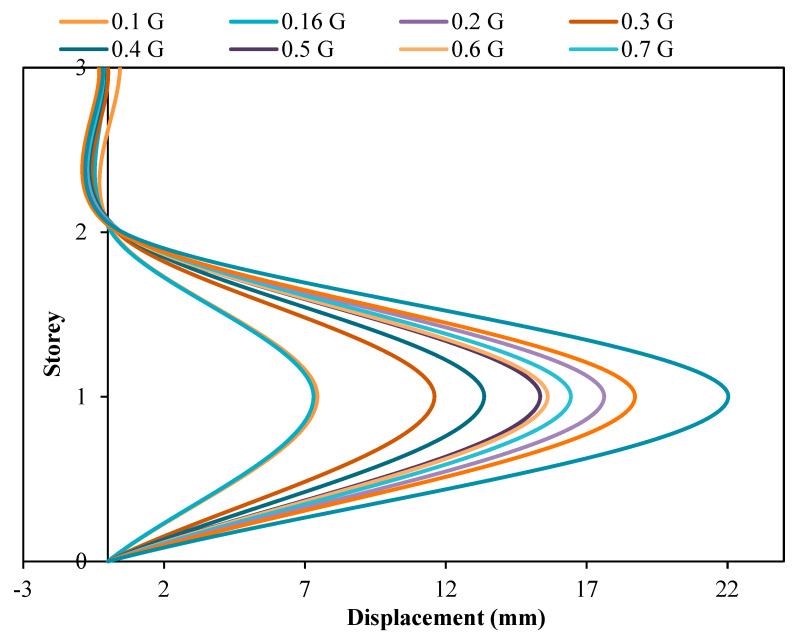
Inter-storey drift of structure with dampers.

Figure 11 and Figure 12, however, show the inter-storey drift of RC structure without and with dampers attached, respectively. Inter-storey drift is the difference in horizontal displacement between the currently selected floor and the one below it. In seismic engineering, inter-storey drift provides clearer insight into the damage inflicted upon a structure subjected to seismic loading. Based on the inter-storey drifting figures, it can be noted that the model with viscous dampers attached shows a reduction in inter-storey drifting at the second storey of the structure when compared with the model without viscous dampers attached. In fact, the maximum reduction of inter-storey drifting at the second floor is 88.32% when the structure is subjected to PGA 0.16 g. On the contrary, the minimum reduction in inter-storey drafting at the second storey is also as high as 40.83% when the structure is subjected to PGA 0.7 G. With the installation of viscous dampers on the three-storey RC structure, inter-drifting at the intermediate storey can be reduced within the approximate ranges of 41% to 88%, when the structure is exposed to PGA 0.1 g to 1.0 g. In addition, both RC structures tested recorded a maximum inter-storey drift at the roof storey of the structure. Thus, it is said that the base storey of the model is vulnerable to structural damage during an earthquake event. The structural failure location was found to be similar to the IBS column performance research conducted by other researchers [56]. This validated that this mode of failure is in line with the current tested seismic structural behaviour results. 

### 3.3. Comparison of Structural Reliability between Model with and without Attached Viscous Dampers

**Figure 13:**
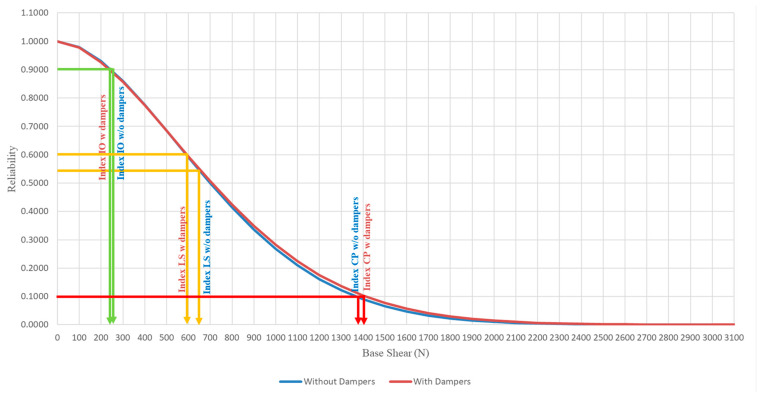
Reliability plot for structures with and without dampers attached to the base storey.

**Figure 14:**
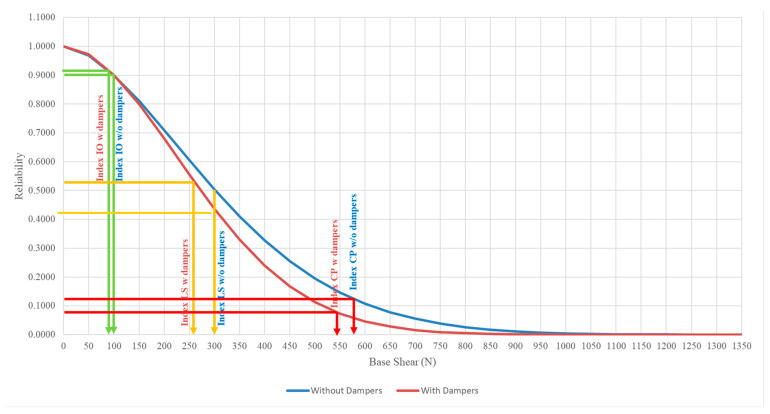
Reliability plot for structures with and without dampers attached on the second storey.

**Figure 15:**
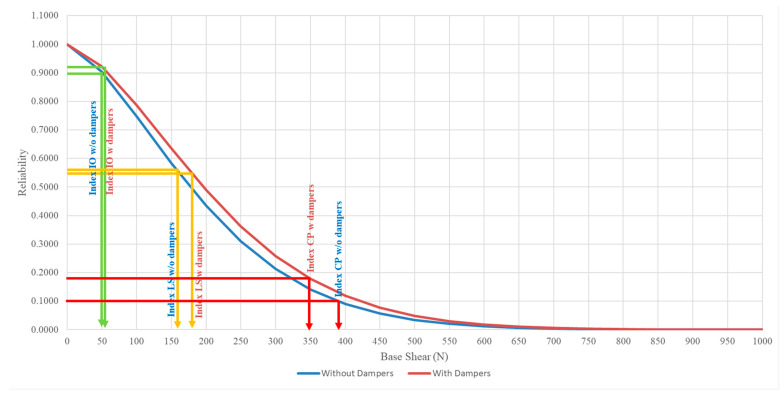
Reliability plot for structures with and without dampers attached to the roof storey.

Figure 13, Figure 14 and Figure 15 show the reliability plot for structures with and without dampers attached at the base, second, and roof storeys, respectively. From the reliability plots, it can be noted that the structural reliability decreases as the base shear increases. This is logical as higher seismic force induced upon the structure will cause more damage to the structure, hence, increasing the chances of failure as PGA increases. The structural performance levels, such as IO, LS, and CP measurements, are based on the Weibull reliability statistical analysis. The reliability analysis for each seismic performance level was divided into 90%, 50%, and 20% thresholds to measure the structural integrity. Although both models subjected to the set of PGAs are categorised under the same performance level, the reliability calculated is slightly different for both models even when the structure experiences the same PGA. For structures without dampers attached, for the base, second, and roof storeys, the RC model values with the IO index are 250 N, 102 N, and 50 N, respectively. For the LS index, the base shear is 699 N, 300 N, and 178 N for the second and roof storeys, respectively. As for the lower boundary limit of the CP index, which also indicates the complete failure of the structure, the base shear capacity of the base, second, and roof storeys of the RC model are 2900 N, 1350 N, and 950 N, respectively. As for the structure with dampers attached, the shear force of the IO index at the base, second, and roof storeys is 234 N, 101 N, and 58 N, respectively. For the LS index, the base shear is 708 N, 273 N, and 196 N for the base, second, and roof storeys, respectively. As for the lower boundary limit of the CP index, the base shear has the capacity of 3100 N, 1050 N, and 1000 N for the base, second, and roof floors as well. 

The model with dampers shows a higher base shear capacity compared to the model without dampers attached. This is because, with the installation of the viscous dampers in the RC model, the viscous dampers will absorb the seismic energy induced upon it and convert them into another form of energy through the compressible liquid contained within the viscous dampers, thus, showing higher resistance in withstanding seismic loading. Table 5 shows the summary output of the statistical analysis, with improvements in the base shear capacity of the model with and without viscous dampers. The reliability shear capacity of the LS index for the model with dampers at the base storey increased from 699 N to 708 N, showing a 1.29% increment, as well as 2900 N to 3100 N, which shows an increment of 6.90% for the CP index. The shear force on the roof storey with the IO, LS, and CP indices increased from 50 N to 58 N, 178 N to 196 N, and 950 N to 1000 N, respectively, which shows the reliability of the rooftop increased by 16% in shear capacity with the IO index, increased by 10.11% in the LS index, and increased by 5.26% in the CP index. The performance of viscous dampers on the second storey remains unchanged. Moreover, this viscous damping system can be installed on to existing building for practical applications along with bracing systems to improve the structural seismic resistance.

## 4. Conclusions

The findings show the various intensity values of PGA affect spectral acceleration. The storey drift and inter-storey drift of the structure were compared and discussed along with the model’s effectiveness. The second finding regarding spectral acceleration showed the relationship between PGA and storey spectral acceleration is directly proportional. This is because storey mass can amplify storey spectral acceleration as the height increases. With the aid of viscous dampers, the maximum reduction of spectral acceleration at the topmost storey was 9.66% (from 2.7661 m/s^2^ to 2.4990 m/s^2^) when the structure is subjected to PGA 0.2 g. The reduction can also be seen when the structure is under the influence of PGA 0.15 g, 0.3 g, and 0.4 g. As for the storey and inter-storey drift, significant reductions in the storey drift and inter-storey drift were recorded as 4.85% at PGA 1.0 g and 88.32% at PGA 0.16 g, respectively, with the aid of dampers. The findings also show that for the model with viscous dampers attached, the mode shape of the structure at the storey-drift-versus-storey-height plot at PGA values of 0.1 g to 0.9 g changed from a single to double curvature shape with the increase in PGA for effective seismic load distribution within the structure.

This research concludes that the reliability of the structure reduces as PGA generated by seismic load increases. The structure with dampers showed a higher base shear performance capacity than the structure without dampers. The novelty of this research shows the base shear resistance capacity increased from 699 N to 708 N (+1.29%) with the IO index and 2900 N to 3100 N (6.90%) with the CP index. Regarding the second storey, the column shear capacity remained unchanged in the IO, LS, and CP indices. Regarding the roof storey, the storey shear capacity in the IO, LS, and CP indices increased from 50 N to 58 N (+16%), 178 N to 196 N (+10.11%), and 950 N to 1000 N (+5.26%), respectively.

## Figures and Tables

**Table 1:** Qualitative/quantitative seismic performance level [26]. Reproduced with permission from [G. Xu], [Journal of Building Engineering]; published by [Elsevier], [2022].

Performance Level	Damage Level	Qualitative Performance Description	Quantitative Performance Description
Operational (O)	No Damage/Negligible Damage	(1) No permanent drift. (2) Structures retained its original strength and stiffness. (3) Structural elements with minor cracking. (4) All important systems fully operate	Depends on construction materials, structure types and analysis models
Immediate Occupancy (IO)	Minor Damage	(1) No permanent drift. (2) Structures retained most of its original strength and stiffness. (3) Structural elements with minor cracking (4) Fire protection system operable.	Depends on construction materials, structure types and analysis models
Life Safety (LS)	Partial Damage	(1) Little permanent drift. (2) Structures retained some of its original strength and stiffness. (3) Structural elements with cracking (4) Building is beyond economical repair	Depends on construction materials, structure types and analysis models
Collapse Prevention (CP)	Extensive Damage	(1) Large permanent drift. (2) Structures close to losing its original strength and stiffness; is near collapse (3) Structural elements with major cracking	Depends on construction materials, structure types and analysis models

**Table 2:** Physics parameters and chemical composition of Ordinary Portland Cement (OPC) [52]. Reproduced with permission from [Mahzabin, M.S], [Construction and Building Materials]; published by [Elsevier], [2018].

Physical Content	Chemical Content % per Unit Weight
Lime (CaO)	64.64
Silica (SiO_2_)	21.28
Alumina (Al_2_O_3_)	5.60
Iron Oxide (Fe_2_O_3_)	3.36
Sulphur Trioxide (SO_3_)	2.14
Magnesia (MgO)	2.06
Nitrogen (N_2_O)	0.05
Loss of Ignition	0.64
Lime Saturation Factor	0.92
Chemical C3S	52.82
Chemical C2S	21.45
Chemical C3A	9.16
Chemical C4AF	10.2

**Table 3:** Optimal design mix.

w/c = 0.42	Water (mL)	Cement (g)	F.A (g)	C.A (g)	Plasticiser
Total	233	550	511	1086	5.50
1 cylinder	0.37	0.86	0.80	1.71	0.01
9 cylinders	3.29	7.77	7.22	15.35	0.08
+20% wastage	3.95	9.33	8.66	18.41	0.09

**Table 4:** Compressive strength of 7-day-cured cylindrical samples.

Concrete Weight (kg)	Ultimate Compressive Strength (N/mm^2^)	Targeted Compressive Strength at 7-day (N/mm^2^)	Status
3.74	33.45	20	Satisfactory
3.45	31.14		Satisfactory
3.48	32.59		Satisfactory

**Table 5:** Summary output of statistical analysis with (W) and without (WO) dampers attached.

Reliability Level	Base Storey			2nd Storey			Roof Storey		
	Shear Capacity (N)								
	WO	W	+%	WO	W	+%	WO	W	+%
0.9 (IO)	250	234	-	102	101	-	50	58	16.00
0.5 (LS)	699	708	1.29	300	273	-	178	196	10.11
0.0 (CP)	2900	3100	6.90	1350	1050	-	950	1000	5.26

## Data Availability

Data sharing is not applicable.
